# Vaccines for Canine Leishmaniasis

**DOI:** 10.1155/2014/569193

**Published:** 2014-12-31

**Authors:** Faeze Foroughi-Parvar, Gholamreza Hatam

**Affiliations:** ^1^Department of Parasitology and Mycology, School of Medicine, Shiraz University of Medical Sciences, Shiraz 71347-45794, Iran; ^2^Basic Sciences in Infectious Diseases Research Center, School of Advanced Medical Sciences and Technologies, Shiraz University of Medical Sciences, Shiraz 71347-45794, Iran

## Abstract

*Leishmania infantum* is the obligatory intracellular parasite of mammalian macrophages and causes zoonotic visceral leishmaniasis (ZVL). The presence of infected dogs as the main reservoir host of ZVL is regarded as the most important potential risk for human infection. Thus the prevention of canine visceral leishmaniasis (CVL) is essential to stop the current increase of the Mediterranean visceral leishmaniasis. Recently considerable advances in achieving protective immunization of dogs and several important attempts for achieving an effective vaccine against CVL lead to attracting the scientists trust in its important role for eradication of ZVL. This paper highlights the recent advances in vaccination against canine visceral leishmaniasis from 2007 until now.

## 1. Introduction

### 1.1. Search Strategy

In this order the articles published in MEDLINE (using PubMed) and Scopus databases were analyzed to extract all information of researches that have been done from 2007 up till now. Canine leishmaniasis, vaccine and* L. infantum* were the main keywords to find records. Although some records were not available in full text format, the authors tried to purchase the full text copy. This study was designed to open novel attitudes through the CVL and the role of* Leishmania *vaccine in dogs in controlling of human visceral leishmaniasis.

### 1.2. The Importance of Canine Visceral Leishmaniasis (CVL)

Leishmaniasis, a complex disease with important clinical diversities from cutaneous to visceral form, is spreading geographically in all continents, except Oceania [1]. Visceral leishmaniasis (VL) is one of the most important infectious diseases with a worldwide distribution and is endemic in at least 88 countries. The incidence is about 500,000 cases annually [2]. It was also considered as one of the six major tropical diseases by the World Health Organization due to its remarkable impact on global public health [3].

*L. infantum*, the main cause of CVL, was described in 1908 by Nicolle and Comte in dogs [4]. This obligate intracellular protozoan parasite has been described as the agent of the Mediterranean VL too. However, it is not just limited to the mentioned basin and is distributed to the Middle East and Asian countries [5, 6]. Since then by developing the sensitive and specific diagnostic techniques, dogs have been emphasized as the natural reservoir host for VL and they are the most important cause of spreading in human VL and CVL in endemic area [1]. Therefore, CVL is considered as a disease of both veterinary and public health importance. This vector-borne parasitic disease is transmitted through the bite of sand flies of the genera* Phlebotomus* (in the old world) and* Lutzomyia* (in the Americas). Furthermore finding the viable* L. infantum* in fleas and ticks of dogs suggests the possible role of ectoparasites of dogs in CVL dissemination [7–9]. Although visceral leishmaniasis remained patent in many canine hosts, it has emerged in various ranges of clinical manifestations by local or generalized lymphadenomegaly, loss of body weight, liver and spleen enlargement, ocular lesion, epistaxis, onychogryphosis, and lameness [10].

Stray dogs based on their local conditions and the lack of proper preventative actions may potentially play a role in maintaining CVL in endemic areas [11]. In this regard the prevention of the disease is extremely complex but prevention measures have focused much more on control of disease in animals [11]. New epidemiological concerns were raised because of the adoption and transportation of dogs from endemic canine leishmaniasis areas to other places and the spread of infection in human [12–14]. The prevalence of CVL reaches 67–80% in enzootic Mediterranean regions [15, 16]. The spread of CVL among dogs has an effective role in the incidence of the infection in human [17]. The prevalence of infection in dogs might be associated with the heavy infection rate of human disease incidence [18, 19]. So, presence of* L. infantum* infected dogs might be a good indicator for areas with high prevalence of VL [20, 21].

### 1.3. Main Strategies for Control of CVL

Measures, such as use of insecticides, drug treatment, and elimination of the infected dogs, have been used for control of CVL [22]. Unfortunately, chemotherapy was not a successful measure and relapsing cases were often seen among treated dogs [23]. In addition, sometimes drugs do not lead to inhibition of infectivity to sand flies [24]. Similar to the symptomatic reservoirs, asymptomatic ones can also play an important role as a reservoir host for transmission of the parasite. Besides, elimination of the infected dogs is ethically unacceptable. Considering the unsuccessful control strategies, establishment of efficacious control ways such as vaccination has a dramatic role in controlling the parasitic infection [25, 26]. Induction of protective anti-*Leishmania *immunity response in dogs is a feasible, important, and cost-effective goal which highly affects control of human leishmaniasis [26].

In addition, there are several reports of feline leishmaniasis caused by* L. infantum *but more studies are needed to confirm the role of cats as reservoir in the nature [23, 27]. There are also some reports of dermotropic* L. infantum *inwhich their transmission from animals to human should be considered by specific projects [28]. Some investigators reported on visceralization of* L. tropica. *So the study of their transmission from dogs to human should be recommended [29]. On the other hand, the coinfection of* Leishmania *and human immunodeficiency virus (HIV) was a serious human threat in the last decades of the 20th century [2]. In recent decades, there are reports about the increasing of ZVL in endemic foci and it seems that the coinfection of visceral leishmaniasis with emerging immunosuppressive conditions in human (e.g., HIV/AIDS) has complicated the issue of CVL [11].

The ideal vaccine includes several molecules which are preferably conserved among different species and expressed abundantly in the tissue amastigote stage, but few antigens can protect against more than one species in animal models. Immune responses have been accompanied with IFN-*γ* and TNF-*α* production by activating macrophages and killing the intracellular parasites associated with protective responses in dogs [30]. Antileishmanial cellular and humoral immunity in dogs have been recognized as distinct indexes in susceptibility/resistance in visceral leishmaniasis infected dogs [31, 32]. With this brief introduction, we can conclude that, to access a convenient and efficacious method for control of ZVL, vaccines could be the best choices.

In the last decades, major activities have been accomplished towards understanding immune mechanisms in canine leishmaniasis and innovating of new vaccines. These candidates are included live parasite (first generation), purified* Leishmania *antigens or live recombinant bacteria expressing* Leishmania *antigens (second generation), and plasmid DNA encoding antigens as third generation [33].

## 2. First Generation Vaccines

Despite the development of* Leishmania *vaccine, injection of live* Leishmania *sp. still remained one of the most effective methods to generate a powerful protection. These could be regarded as one of the most commonly used canine* Leishmania* vaccines too. Attenuated live parasite vaccines can be obtained through culturing in different* in vitro* mediums [34] using temperature sensitivity [35], gamma irradiation [36], or chemical mutagenesis [37]. However, leishmanization has been abandoned in man, and parasite transgenesis was successfully used in order to generate immunogenic but attenuated organisms. In this matter a variety of parasites with attenuated virulence genes could be produced. The selected target gens for attenuation are usually responsible for encoding virulence factors (or their synthetic enzymes) and metabolic pathway components. In addition some scientists have been used transgenic parasites that secrete host immune mediators to boost anti-parasite responses and facilitate parasite clearance [38].

Daneshvar et al. compared inoculation of gentamicin attenuated* L. infantum*, culturing* L. infantum* promastigotes under pressure of gentamicin, to wild type parasites. They characterized the immunophenotypic profile of mononuclear cells of immunized dogs with the attenuated promastigotes of* L. infantum* cocultured with 10% gentamicin. The percentage of CD4^+^ and CD8^+^ T cells in dogs vaccinated by gentamicin attenuated parasites was higher than control ones. In addition no parasite was reported in the polymorphonuclear cells of animals that received gentamicin attenuated promastigotes, whereas promastigotes parasites were seen in polymorphonuclear cells of 60% of animals that received wild type of* L. infantum* as vaccine. Groups were maintained in indoor places with windows covered by deltamethrin sprayed. No clinical signs or biochemical and hematological abnormalities were found in any of the dogs. No clinicopathological changes were observed during 12-month followup in dogs. They concluded that the attenuated line is capable of protecting dogs against wild type* L. infantum* [39]. In a similar study by Daneshvar et al., 14 healthy mixed breed dogs were immunized with these attenuated strains, and the level of IFN-*γ* in gentamicin attenuated ones was reported to be higher than the control ones. No DNA of parasite was detected in BM aspirate of immunized animals 12 months after infection [40]. A vaccine formulation against CVL consisted of centrin deleted* L. donovani *parasite (Ld Cen−/−), applied to healthy dogs. This deletion specifically affected the profile of the amastigote stage that replicates inside the macrophages. Animals were studied in three groups. Those were injected subcutaneously by Ld Cen−/−* L. donovani *promastigotes at stationary phase. Another group received Leishmune (commercial vaccine). The third group received just PBS. Level of cytokine and antibody production and proliferative lymphocyte responses were measured 15 days after the injection of vaccines. Ld Cen−/− vaccinated group exhibited higher antibody titer compared to the Leishmune vaccinated group. Besides, higher T and B cell proliferation were induced upon stimulation with Ld Cen−/− vaccine which generates the type 1 T helper cells/type 2 T helper cells balance in order to reach a protective response. The increase of TNF-*α* and IL-12 was recorded in supernatant of cultures, supporting the fact that Ld Cen−/− live attenuated vaccine has immunogenic and protective effects against VL in dogs [2]. The safety of Ld Cen−/− has been described earlier in mice and hamsters [41].

## 3. Second Generation Vaccines

Recent advances in the design of second generation vaccines against canine leishmaniasis using various strains of dogs have shown that immunization with defined parasite antigens provides protection against challenge with* L. infantum.* This generation includes whole crude antigen of cultured parasite, the native fractions purified from the parasites based on abundance and surface localization or recombinant antigens created by genetic engineering which stimulate the immune response in the best way. These kinds of* Leishmania* vaccines are organized as second generation vaccines [42].

### 3.1. Whole Crude Antigens of Parasites

Based on Roatt et al. effort in Brazil, 20 dogs in four separate groups, respectively, were injected subcutaneously by PBS,* L. braziliensis* promastigote protein, saponin, and* L. braziliensis* promastigote protein and saponin 3 times during 4 weeks. After 105 days animals were challenged intradermally by late log phase of* L. infantum* promastigotes and salivary gland extract of* Lutzomyia longipalpis*. Animals were followed 885 days after challenge.* L. braziliensis *crude antigen plus saponin vaccine (LBSap) is capable of inducing significant humoral immune response with increase of specific anti-*Leishmania* IgG and the subclasses (IgG1 and IgG2) after intradermal inoculation of* L. infantum *and salivary gland extract of* L. longipalpis*. LBSap vaccine promoted a mixed profile of immune response. Increased level of T4 cells as well as T8 cells was observed in simultaneous humoral responses. Interestingly the increased level of IFN-*γ*, the biomarker of immunogenicity and protection against* Leishmania*, was shown in the spleen cells of dogs; real-time PCR showed significant reduction of parasite load in spleen. Altogether, this study claimed that LBSap vaccine is highly immunogenic and depicts persistent humoral and cellular immune response against canine* L. infantum *infection [43]. In a similar study* L. braziliensis* promastigote proteins and saponin as adjuvant (LBSap) induced a long-lasting (885 days after* L. chagasi* challenge) type 1 immune response against* L. chagasi *challenge infected dogs. This immunity was accompanied by high production of IFN-*γ* and IL-12 [44, 45].

### 3.2. Fraction Purified Parasites

Many of these protective molecules are located on the surface of parasite. In this context diverse studies have been carried out in order to establish immunity in dogs. Nevertheless, two second generation vaccines are commercially available in Brazil (Leishmune and Leish-Tec); more trials are in progress in this area [26].

In one study, about 400 seronegative healthy dogs without a previous history of CVL received excreted/secreted antigens purified from the culture supernatant of* L. infantum *promastigotes (LiESAP) with muramyl dipeptide (MDP) as adjuvant. This vaccine has been licensed recently in Europe (CaniLeish, Virbac Animal Health). All dogs were kept outdoors, exposed to sand flies in an endemic region of south of France. Vaccinated dogs' antibody reactivity to the antigen by immunofluorescence antibody (IFA) test showed a strong reaction after two seasons of sand flies activity. Moreover, strong vaccine efficacy (92%) was seen based on leishmanial DNA and parasite detection in bone marrow aspirate. Besides, the* in vitro* study of T cells (Th1 type) and their responses were confirmed by high production of IFN-*γ* in the supernatant of the cocultured cells from the vaccinated dogs in comparison to the control ones [46].

Another study in France reported that use of LiESAP-MDP vaccine could protect dogs against the parasite for 2 or 8 months after the vaccination. Significant and long-lasting protection in correlation with an early Th1-type cellular immune response was seen in vaccinated group. In addition, an exclusive increase in IgG2 antibodies to LiESAP was observed in the test group at the second 8th month after infection [47].

Leishvaccine (including* L. amazonensis *strain (IFLA/BR/1967/PH8)) and Leishmune (composed of lyophilized* L. donovani *purified fucose mannose ligand (FML)) were applied to vaccinate 24 healthy dogs in Brazil. 12 dogs in each group (Leishvaccine and Leishmune) were inoculated subcutaneously by 3 vaccine doses plus BCG as adjuvant every 21 days. Araújo et al. observed that Leishvaccine and Leishmune initiated a clear immunophenotypic variation in the innate immune response. They also showed that Leishmune was a selective stimulator of phagocytes. Leishmune and Leishvaccine could be considered as significant vaccines with a high immunological potency against CVL. This study showed that Leishvaccine and Leishmune developed a distinct immunological profile in dogs. Furthermore, a mixed cytokine pattern with increased level of IFN-*γ* and IL-4 was detected in Leishvaccine group. Leishmune vaccine elicited more characteristic cytokine pattern by well-improved IFN-*γ* and NO concentration mixed (IFN-*γ* and IL-4) cytokine pattern; upper levels of anti-*Leishmania* IgG1 indicate immunobiological enhancement of T cells. This fact emphasizes the substantial role of these vaccine formulations in control of CVL [48]. According to Araújo et al. publication in 2008, 24 German dogs were divided into two groups. One group was immunized with 3 subcutaneous doses of Leishvaccine (consisting of* L. amazonensis *(strain IFLA/BR/1967/PH8)) plus BCG as adjuvant every 21 days. Another group received Leishmune (consisting of* L. donovani *purified FML). Leishvaccine was accompanied with activation of T cells and B lymphocytes. Early activation of CD4^+^ T cells (CD4^+^ MHCII) and a later activation of B cells (CD 32 in B cells) and CD8^+^ T cells (CD8^+^ CD18^+^) were considered. Leishmune included a preferential involvement of CD8^+^ T cells, with no phenotypic changes in circulating B lymphocytes, indicating the fact that a selective pathway as a result of cooperation between antigen-presenting cells (APCs) and cytotoxic T cells has occurred. The purified nature of FML antigen in Leishmune confirmed a more selective activation of innate immunity cells. On the other hand, the whole crude* L. amazonensis *antigen (Leishvaccine) created broader phenotypic changes. However, both vaccines showed a pivotal role in triggering innate immunity cells and could be considered priority vaccines with a high quality immunogenic potential against CVL [49].

In another attempt, 30 dogs were vaccinated with FML (Leishmune) in three monthly doses. They received two annual booster doses and were investigated 3 to 5 months after the last booster in order to find anti-*Leishmania *antibodies in a VL endemic area. Simultaneously, 30 dogs that had not been vaccinated with Leishmune were naturally infected by* L. chagasi* as control group. It was shown that, despite detection of* Leishmania *in skin by using PCR, specific anti-*Leishmania* IgG is determined by FML-ELISA in vaccinated groups. These findings were regarded as the Leishmune parasitological protection after vaccination [50]. In another similar story, Leishmune was tested on 20 dogs. Animals were injected subcutaneously by Leishmune at 21 intervals. Ten days and 6 and 12 months after the last vaccine dose blood and bone marrow samples were collected. At the same time placebo group consisting of 20 dogs were injected by placebo and followed throughout the study. This vaccine showed 100% humoral responses against parasite. Cellular response (85%) was conferred in the supernatant of cultured cells stimulated with FML [48, 51].

Moreover, in a similar project, 172 dogs were studied in 4 groups as follows. Group 1 consisted of 45 healthy dogs that were kept in a nonendemic area for CVL as negative control. Group 2 consisting of 45 dogs were naturally infected by* Leishmania *sp. and CVL symptoms appeared in them. Group 3 consisted of 45 asymptomatic dogs naturally infected by* Leishmania *sp. Group 4 consisted of 37 healthy dogs vaccinated by Leishmune in 3 doses every 21 days. Serological tests, lymph nodes, and bone marrow aspiration were performed during 6-month follow-up study. The first peak of antibodies was detected 48 days after the last vaccine dose. Vaccinated dogs (group 4) showed a boosting and then decreasing of antibody responses. High levels of antibody were seen 138 days after the last booster. And up to 6 months after Leishmune vaccination antibody peak was observed in vaccinated animals. This study indicated that antibody production in vaccinated dogs and positivity up to 6 months may identify vaccinated animals as naturally infected dogs [52]. In this context, at least two different serological methods (indirect immunofluorescence antibody (IFAT), counterimmunoelectrophoresis (CIE), or direct agglutination test (DAT)) were recommended for detection and improvement of specificity and sensitivity of laboratorial canine visceral leishmaniasis. Furthermore, lymph node aspirates were considered as the least invasive and the most appropriate sample for diagnosis of visceral leishmaniasis [53].

The P-8 antigen, another purified fraction, was refined from the surface membranes of axenically cultured* L. pifanoi *amastigotes which were isolated by using nitrogen cavitation and differential centrifugation [54, 55]. In this project,* L. pifanoi *P-8 antigen induced a 3-4-fold higher level of IFN-*γ* expression in comparison to* L. infantum *soluble antigen in 3- to 4-month-old female dogs challenged by* L. infantum *or* L. chagasi *parasites. Also, a higher expression of TNF-*α* was found in P-8 antigen group compared to the soluble* Leishmania *antigen (SLA) group. Therefore, with regard to the higher lymphoproliferative response and level of IFN-*γ* expression induced by P-8, Carrillo et al. suggested that this antigen might be a potential vaccine candidate for controlling CVL [56].

### 3.3. Recombinant* Leishmania* Antigens

Recombinant* Leishmania *antigens, as another division of second generation vaccines, could be considered as available and cost saving vaccination methods. These antigens are able to produce strong but short-lived protections. Several* Leishmania* genes have been used in various projects. They could be delivered as bacteria manufacturing purified proteins and naked DNA (as the more advanced step). Injection of these kinds of antigens may have the advantage of adjuvant effects which may stimulate antigen-presenting cells [57].

The recombinant* L. infantum *histones H1 and hydrophilic acylated surface protein B1 (HASPB1) were administered individually or as a cocktail in combination with Montanide TM ISA 720 adjuvant in 48 beagle dogs. The* L. infantum* H1 was cloned into the pGEX-KG vector and then expressed in* Escherichia coli. *HASPB1 was cloned into pET15b vector and expressed in* E. coli*. The purification method in both antigens was different too. Histone H1 was purified using resin GST affinity resin but the HASPB1 was purified using Ni-NTA and anion exchange column. Forty eight dogs were divided into 7 groups. H1, HASPB1, and H1 + HASPB groups were inoculated by 3 intradermal vaccine doses monthly. Other groups included adjuvant (MML and Montanide) and control groups. 45 days after the last vaccine booster dogs were infected with* L. infantum* promastigotes. In this study the cocktail antigenic vaccines (H1 + HASPB1) were able to induce a variable partial protection while histone H1 antigen alone produced stronger immunization among dogs. Parasite burden reduction, even though partial, was also detectable in the bone marrow and lymph nodes of group HASPB1 + H1 vaccinated dogs compared to the control group [58]. In another work in Madrid, Spain, the dogs were immunized with three recombinant* Leishmania *antigens: heat shock protein- (HSP-) 70, paraflagellar rod protein- (PFR-) 2, and kinetoplastid membrane protein- (KMP-) 11 as routine vaccination protocol. Animals were followed up after the experimental* L. infantum* challenge during a 1.5-year period. Under the condition used in this study and due to the moderate Th1 response in HSP-70, KMP, and PFR vaccinated dogs, the IFN-*γ* mRNA expression was increased in all PBMC from mentioned recombinant antigens. It could be suggested that these recombinant antigens play a probable role in the protection against CVL [59].

The chimerical protein “Q” consisted of five antigenic fragments of acidic ribosomal proteins (Lip2a, lip2b, and Lip2b Po) and histone H2A protein. It was designed from* L. infantum *rJPCM-strain sequences (“rJPCM5_Q”) and tested in two groups of dogs in combination with different adjuvants. Groups were designed based on the difference between the gene expression methods. Gene sequence in the first group was expressed in the* Baculovirus* system (consisting of 28 dogs) and in the second group (consisting of 16 dogs) it was expressed in* E. coli. *Live BCG, muramyl dipeptide (MDP), aluminum hydroxide Al (OH), matrix C, and killed* Propionibacterium acnes* were used as adjuvant in both* Baculovirus* and* E. coli*-produced recombinant antigen. Animals received two subcutaneous vaccine doses with 3-week intervals. Three to four weeks after the final dose, animals were infected experimentally with stationary phase of* L. infantum* promastigotes. Sampling was done weekly during 10-month follow-up period. All dogs except one were reported positive for parasite culturing of bone morrow and lymph node aspiration biopsies. Nonetheless, none of the candidate vaccines prevented the establishment of the parasite or promotion of the clinical manifestations. Both groups either* Baculovirus* or* E. coli* produced JPCM5 Q protein induced cellular immune responses after vaccination. Those groups received antigen plus Al (OH) and antigen plus ISCOMatrix in comparison to the control ones which showed significant cellular proliferation. Because of single control dog in* Baculovirus* gene produced group, evaluation could not show clear effect. The failure point was related to the differences in the adjuvant and this study claimed that although bacillus Calmette-Guérin (BCG) is not an appropriate adjuvant for canine vaccines in view of the local reactions, it had a strong adjuvant effect in combination with Q protein that could promote a significant protective immunity against* L. infantum *CVL [60].

In another study, 22 young uninfected dogs were enrolled for vaccination with recombinant modified virus Ankara (MVA) expressing tryparedoxin-peroxidase (TRYP) and* Leishmania *activated C kinase (LACK). Animals randomly received two intramuscular injections on days 0 and 28. The vaccinated animals showed higher antigen-specific antibody levels. Moreover, those who had received DNA/MVA TRYP produced a type 1 dominated proinflammatory cellular immune response compared to the DNA/MVA-LACK receiving group. In other words, DNA/MVA induced both cellular immunity and humoral immunity. Also this study suggested that cell immunity was prolonged for at least 4 months after vaccination in the absence of restimulation or natural infection. Actually field trials need to confirm the effect of DNA/MVA TRYP vaccine in prevention of CVL [61].

In the southern area of Italy, 3 groups of dogs (each group: 15) were injected subcutaneously: first group by MML (multisubunit recombinant* Leishmania*) polyprotein plus MPL as adjuvant, second group by MML plus adjuvant, and third group by saline monthly in 3 doses. Animals were exposed to sand flies bites. One year later, after the last vaccine dose and before the transmission season, surviving dogs received another three-dose vaccine profile. The anti-MML IgG antibodies were decreased after the second and third steps of vaccination and after the end of the 2-year study 95% of vaccinated dogs showed leishmanial infection. They confirmed that the MML vaccine was not capable of effective protection at that area, either from natural* Leishmania* infection or from disease progression [62]. However, MML vaccine has exhibited protective immunity against* L. infantum* in mice and hamsters in another study [63].

Recombinant A_2_ antigen (Leish-Tec), an amastigote specific antigen ranging from 45 to 110 kDa, formulated as a vaccine, could develop type 1 immune responses. 14 animals were immunized subcutaneously by A_2_ recombinant protein plus saponin as adjuvant; just 7 dogs were challenged by* L. chagasi* promastigotes. There were two groups that received saponin and PBS, respectively, as control groups. Vaccination profile was included: injecting on days 0 and 21 of the study and challenging 4 weeks after the last vaccine dose. Increased levels of total IgG and IgG_2_ were produced in vaccinated animals. Also, detection of high levels of IFN-*γ* in vaccinated animals compared to the control ones confirmed the protective effect of recombinant A_2_ protein plus saponin [64].

The* Leishmania*-derived recombinant polyprotein Leish-111f which included three component proteins (thiol-specific antioxidant (TSA),* L. major* stress-inducible protein 1 (LmSTI1), and* Leishmania* elongation initiation factor (LeIF)) is a subunit vaccine that has been demonstrated to be safe in human clinical trial [65]. In another study, Leish-111f formulation with monophosphoryl lipid A in stable emulsion (MPL-SE) as adjuvant had accelerated the cure of visceral leishmaniasis in active natural CVL. This vaccine was reported to be efficient in mild cases of CVL [66].

## 4. DNA Vaccines

New approaches toward finding appropriate DNA vaccines illustrated a desirable policy to prevent the transmission of* Leishmania *from dogs to other mammalian hosts. In DNA vaccines, there is no need for cold chain and so they have steady effects in experimental models and a lot of studies are going on in laboratories for development of DNA vaccine against CVL [67]. Through this process, while a foreign antigen is expressed in the plasmid DNA, it can lead to strong antibody production as well as complete cell mediated immune responses. These kinds of* Leishmania* vaccines induced extensive cellular response and more efficient protection. Literature has shown that humoral and both CD4 and CD8 T cell mediated immune (CMI) responses were elicited, and long-lasting immunity was achieved through immunization [68].

Intradermal DNA immunization of healthy dogs with the cocktail of four different plasmids encoding for* L. infantum* Gp63 (major surface glycol protein), LACK (*Leishmania* activated C kinase), KMP-11 (kinetoplastid membrane protein 11), and TRYP (tryparedoxin-peroxidase) did not lead to a satisfactory outcome. Dogs received 4 doses every 15 days and 1 month after the last booster they were injected intravenously by* L. infantum* promastigotes. Although the multiantigenic plasmid DNA vaccine was safe and well tolerated by all 12 dogs, most of them showed patent clinical signs during 3 to 4 months after infection. No significant differences were observed between the vaccinated animals and the control group regarding the serological tests and CMI responses. In other words, the high level of antibody concentration with high amount of DNA parasites in lymph nodes, liver, and spleen suggested unprotective effects of vaccine [69].

In another attempt, two forms of DNA-LACK vaccine (recombinant vaccine virus-LACK (rVV-LACK) and modified virus Ankara-LACK (MVA-LACK)) triggered Th1-type immune response in 8 dogs. Animals were studied in two groups. The ones that were subcutaneously injected by DNA-LACK received recombinant vaccine (rVV-LACK) virus after the 15 days as the second dose. Another group received as a first dose DNA-LACK and 15 days later was boosted by modified virus Ankara (MVA-LACK) in subcutaneous form. All dogs were challenged experimentally by* L. infantum* promastigotes 2 weeks later. Less clinical symptoms of CVL were seen in group DNA-LACK/MVA-LACK, relating the fact that boosting with nonreplicative virus confers better protection. Besides parasites DNA in target organs (liver and spleen) showed high DNA quantity in control group compared to both vaccinated groups. This DNA vaccine was able to establish protection associated with the absence of leishmaniasis symptoms (62.5% of cases in vaccinated groups were seen asymptomatic after 290 days), lower level of* Leishmania*-specific IgGs (compared to control group), higher amounts of T cell activation, and increase of Th1 cytokine synthesis (IFN-*γ* and IL-12) [70].

## 5. Conclusion

Due to the risk of imported infected dogs, CVL is considered a global and also veterinary concern today. Furthermore, immunization in dogs could control the disease among humans in endemic area. Based on different means and approaches discussed in this paper, prominent development of CVL vaccines will be expected in the future. Considering the broad range of antigen markers with vast species coverage, it is essential to access greater scientific information about canine immunology for developing and creating a convincing and acceptable CVL vaccine. Studies showed that first generation vaccines have inadequate ability to produce long-lasting immunity. The induced immunogenicity by these vaccines was not carried over to protective effect. In addition, third generation vaccines are protective but due to the varied nature of DNA antigens, the true impacts of mentioned vaccines are unclear. So the second generation of vaccines could be one of the best choices for canine* Leishmania* vaccination. To find a commercial canine vaccine, Brazil was the first country to license the commercial Leishmune vaccine in 2003. Leishmune consisted of* L. donovani* glycoprotein (fucose mannose ligand) with saponin as adjuvant. Leish-Tec vaccine composed of the A2 antigen (a recombinant protein from amastigote stage of different* Leishmania* species) plus saponin was registered in 2007 by the Brazilian Ministry of Agriculture [71]. Advances achieved in CVL vaccines could play a noticeable role in the control of human VL.
